# Molecular Insights into α-Synuclein Fibrillation: A Raman Spectroscopy and Machine Learning Approach

**DOI:** 10.1021/acschemneuro.4c00726

**Published:** 2025-01-28

**Authors:** Nathan P. Coles, Suzan Elsheikh, Agathe Quesnel, Lucy Butler, Claire Jennings, Chaimaa Tarzi, Ojodomo J. Achadu, Meez Islam, Karunakaran Kalesh, Annalisa Occhipinti, Claudio Angione, Jon Marles-Wright, David J. Koss, Alan J. Thomas, Tiago F. Outeiro, Panagiota S. Filippou, Ahmad A. Khundakar

**Affiliations:** † School of Health & Life Sciences, Teesside University, Middlesbrough TS1 3BX, United Kingdom; ‡ National Horizons Centre, Teesside University, Darlington DL1 1HG, United Kingdom; § School of Computing, Engineering & Digital Technologies, Teesside University, Middlesbrough TS1 3BX, United Kingdom; ∥ Centre for Digital Innovation, Teesside University, Middlesbrough TS1 3BX, United Kingdom; ⊥ Biosciences Institute, Cookson Building, Framlington Place, Newcastle University, Newcastle upon Tyne NE2 4HH, United Kingdom; # Division of Neuroscience, School of Medicine, University of Dundee, Nethergate, Dundee DD1 4HN, Scotland; ∇ Newcastle Biomedical Research Centre, Newcastle University, Newcastle upon Tyne NE2 4HH, United Kingdom; ○ Translational and Clinical Research Institute, Faculty of Medical Sciences, Newcastle University, Newcastle upon Tyne NE2 4HH, United Kingdom; ◆ Department of Experimental Neurodegeneration, Center for Biostructural Imaging of Neurodegeneration, University Medical Center, Göttingen 37077, Germany; ¶ Max Planck Institute for Multidisciplinary Sciences, Göttingen 37077, Germany; ⋈ Deutsches Zentrum für Neurodegenerative Erkrankungen (DZNE), Göttingen 37077, Germany; ⧓ Laboratory of Biological Chemistry, School of Medicine, Faculty of Health Sciences, Aristotle University of Thessaloniki, 54124 Thessaloniki, Greece

**Keywords:** α-synuclein aggregation, Lewy body diseases, Raman spectroscopy, machine learning analysis, β-sheet formation, fibrillation pathway

## Abstract

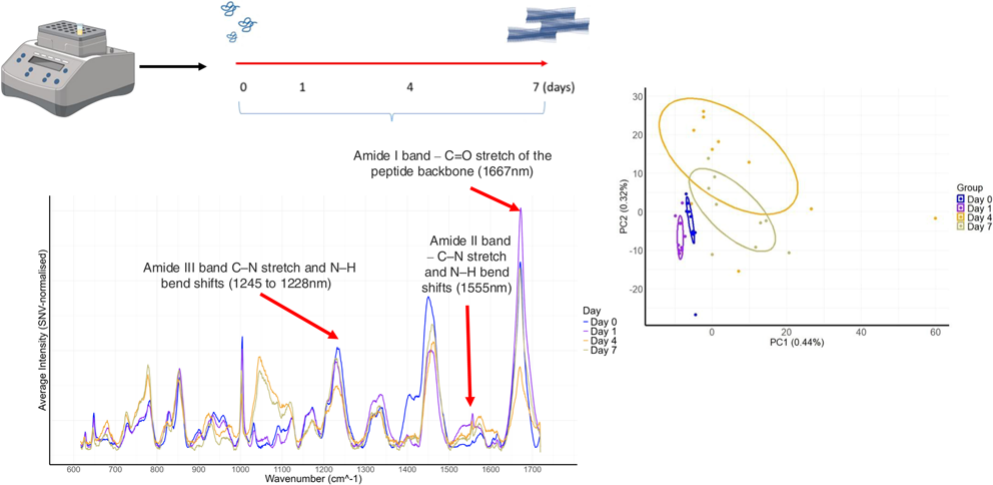

The aggregation of α-synuclein is crucial to the development of Lewy body diseases, including Parkinson’s disease and dementia with Lewy bodies. The aggregation pathway of α-synuclein typically involves a defined sequence of nucleation, elongation, and secondary nucleation, exhibiting prion-like spreading. This study employed Raman spectroscopy and machine learning analysis, alongside complementary techniques, to characterize the biomolecular changes during the fibrillation of purified recombinant wild-type α-synuclein protein. Monomeric α-synuclein was produced, purified, and subjected to a 7-day fibrillation assay to generate preformed fibrils. Stages of α-synuclein fibrillation were analyzed using Raman spectroscopy, with aggregation confirmed through negative staining transmission electron microscopy, mass spectrometry, and light scattering analyses. A machine learning pipeline incorporating principal component analysis and uniform manifold approximation and projection was used to analyze the Raman spectral data and identify significant peaks, resulting in differentiation between sample groups. Notable spectral shifts in α-synuclein were found in various stages of aggregation. Early changes (D1) included increases in α-helical structures (1303, 1330 cm^–1^) and β-sheet formation (1045 cm^–1^), with reductions in COO^–^ and CH_2_ bond regions (1406, 1445 cm^–1^). By D4, these structural shifts persist with additional β-sheet features. At D7, a decrease in β-sheet H-bonding (1625 cm^–1^) and tyrosine ring breathing (830 cm^–1^) indicates further structural stabilization, suggesting a shift from initial helical structures to stabilized β-sheets and aggregated fibrils. Additionally, alterations in peaks related to tyrosine, alanine, proline, and glutamic acid were identified, emphasizing the role of these amino acids in intramolecular interactions during the transition from α-helical to β-sheet conformational states in α-synuclein fibrillation. This approach offers insight into α-synuclein aggregation, enhancing the understanding of its role in Lewy body disease pathophysiology and potential diagnostic relevance.

## Introduction

**Figure 1:**
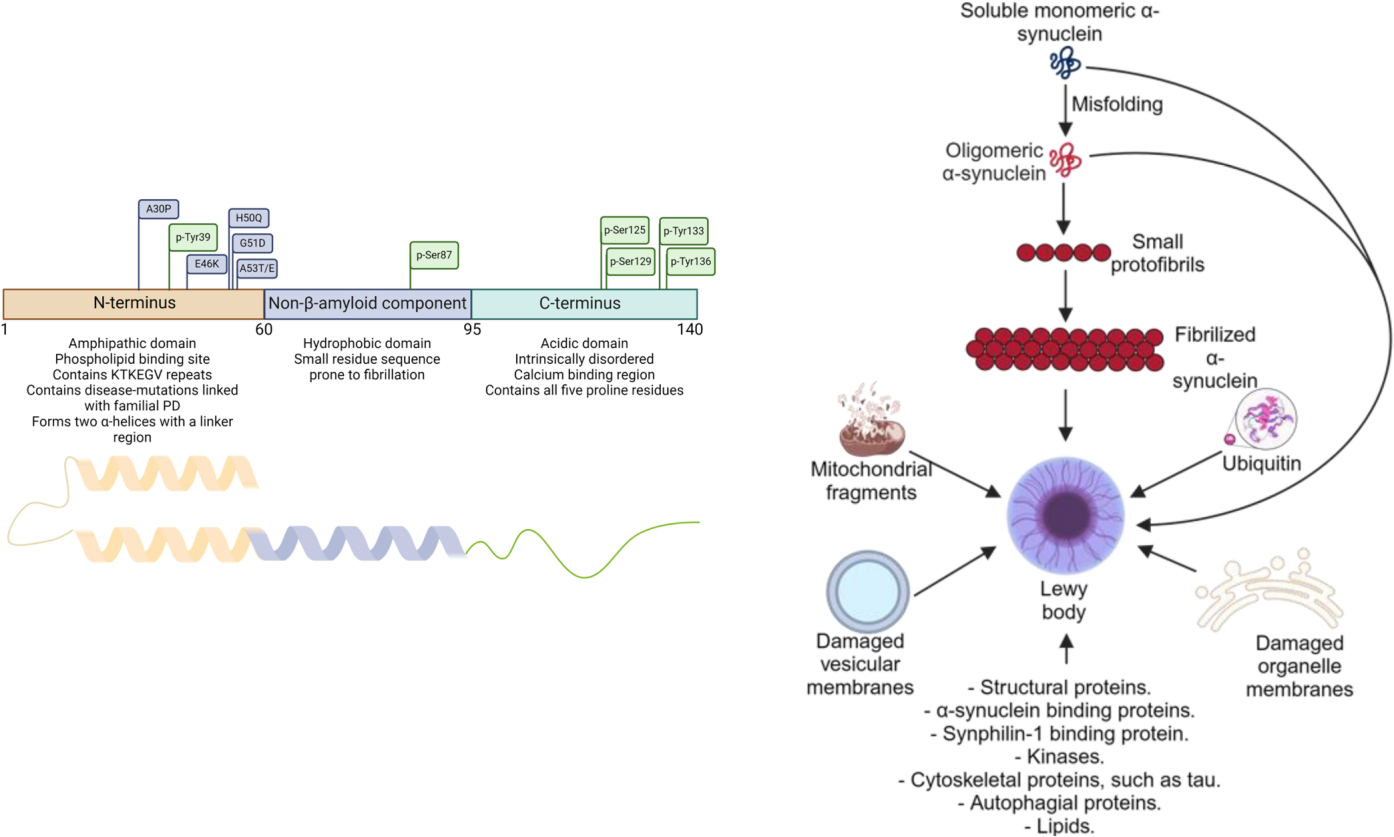
Left panel: Structure, amino acid sequence, and aggregation cascade of α-synuclein. The figure highlights important amino acids linked with familial PD (in blue) and phosphorylation sites (in green). Right panel: Hypothetical aggregation cascade of physiological monomeric α-synuclein, which misfolds into toxic oligomers and elongates through the recruitment of additional monomeric α-synuclein. These fibrillar structures, along with various other α-synuclein species, proteins, and damaged cellular components, colocalize to form Lewy bodies (created with BioRender.com).

The aberrant accumulation of α-synuclein is the key defining pathological feature of Lewy body diseases, such as Parkinson’s disease (PD) and dementia with Lewy bodies (DLB).^1−3^ This culminates in the formation of Lewy bodies and neurites, disrupting cellular function and driving neurodegeneration as the protein transitions from a soluble monomer to insoluble fibrils, a hallmark of disease progression.^4−6^ In its native form, α-synuclein is a soluble, monomeric protein with a molecular weight of 14,460.16 Da, composed of 140 amino acids.^7^ Its structure includes three distinct regions: the N-terminal domain (amino acids 1–60), which contains several repeat motifs (KTKEGV) critical for tetramer formation;^8,9^ the central hydrophobic domain (aa 61–95), responsible for β-sheet formation^10,11^ and implicated in protein aggregation;^12^ and the C-terminal domain (amino acids 96–140), which inhibits oligomerization through interactions with the central region^13,14^ (Figure 1A). In its native state, α-synuclein plays a crucial role in vesicle binding, interacting with soluble *N*-ethylmaleimide sensitive factor attachment protein receptor (SNARE) proteins at the synapse.^15,16^ The formation of the SNARE complex, involving t-SNARE and v-SNARE proteins, induces membrane distortion, facilitating vesicle binding for exocytotic release.^17^ The interaction between α-synuclein and vesicle-associated membrane protein 2 (VAMP2), a core component of the SNARE complex, plays a role in neurotransmitter release through the regulation of the vesicular reserve pool.^18^ Nuclear α-synuclein has also been shown to interact with both DNA^19^ and histones,^20^ and has been shown to undergo modifications in DLB.^21^

The aggregation pathway of α-synuclein typically follows a well-defined sequence of nucleation, elongation, and secondary nucleation, with the potential for prion-like spreading.^22−24^ While the latter stages of aggregation are well characterized, the early events of primary nucleation, including dimer formation,^25,26^ remain less well understood. Primary nucleation is proposed to occur via oxidative cross-linking of tyrosine residues, inducing conformational changes that stabilize dimers, which then form oligomers.^27,28^ The fibrillation process can lead to structural changes, including the formation of β-sheets, which are crucial for neurotoxicity.^29^ Secondary nucleation allows new fibrils to form adjacent to existing fibrils, thereby propagating the aggregation process. Cellular environmental conditions significantly influence the structure of α-synuclein fibrils,^30,31^ which form the basis of Lewy inclusions. Lewy bodies, however, also contain other biomolecules, such as ubiquitin and neurofilament proteins,^32,33^ along with lipid biomolecules, dystrophic mitochondria, and other organelles^34,35^ (Figure 1B).

Raman spectroscopy is a technique that probes molecular vibrational states by analyzing the inelastic scattering of photons from a sample. Since the vibrational modes of each molecule are distinct, a molecule can be identified by its characteristic Raman spectrum.^36^ Raman spectroscopy is emerging as an invaluable tool in pathological research, offering detailed molecular insights into diseases like cancer and neurodegenerative disorders, significantly advancing our understanding of disease mechanisms and progression.^37−40^ Such capabilities make Raman spectroscopy an invaluable method for protein characterization, providing label-free, nondestructive insights into structure, dynamics, and microenvironmental influences.^41−43^ This is especially important for understanding disease mechanisms, where protein aggregation plays a central role, as in many neurodegenerative disorders. The integration of machine learning techniques has further enhanced the utility of Raman spectroscopy in differentiating between disease states, emphasizing its potential in clinical diagnostics.^37,40,44^

In this study, we used Raman spectroscopy combined with machine learning to investigate biomolecular changes during α-synuclein fibrillation. Recombinant wild-type α-synuclein was produced, purified, and used in a fibrillation assay to generate preformed fibrils (PFFs). We analyzed Raman spectral peaks at various stages of aggregation to assess structural alterations in the protein, examining the fibrillation process at different time points. Complementary techniques, including liquid chromatography-tandem mass spectrometry (LC-MS/MS), dynamic and static light scattering, and negative staining transmission electron microscopy (TEM), were employed to provide a detailed characterization of α-synuclein fibrillation. By tracking specific Raman spectral peaks throughout aggregation, we aim to reveal critical structural changes in α-synuclein, potentially enhancing our understanding of its role in Lewy body diseases.

## Methods

### Protein Production, Purification, and Fibrillation

BL21 DE3 *Escherichia coli* (Thermo Fisher) were transformed with a pET21a-asyn plasmid for α-synuclein overexpression (courtesy of Professor Outeiro’s laboratory). Competent values were *E. coli* were incubated on ice for 30 min, heat-shocked at 42 °C for 45 s, and returned to ice for 2 min. After adding S.O.C. media, the cells were incubated at 250 rpm at 37 °C for 1 h, plated on LB agar with 100 μg/mL ampicillin, and incubated at 37 °C overnight. Bacterial colonies were picked and cells were grown in liquid cultures at 37 °C with shaking at 225 rpm to an OD600 of 0.6, and 1 mM IPTG was added for 2 h to induce protein production at 37 °C. The cells were pelleted at 5000*g*, and the media was stored at −20 °C for later analysis. The bacterial cell pellet was resuspended in 30 mL lysis buffer (750 mM NaCl, 10 mM Tris-HCl, pH 7.6, 1 mM EDTA) with cOmplete mini protease inhibitor (Roche). The cell extract was ultrasonicated for 5 min in alternating 30 s cycles at 60% power, then boiled at 95 °C for 15 min to precipitate unstructured proteins. α-synuclein remained in the supernatant after centrifugation at 16,000*g* for 20 min. The sample was filtered through a 50 kDa and then a 3 kDa MWCO filter. The 3 kDa filter was inverted, centrifuged to isolate the retentate, and filtered with a 0.22 μm filter. The supernatant was stored at −20 °C. An ÄKTA fast-protein liquid chromatography (Cytiva Life Sciences, Marlborough, MA) system was used to purify the proteins. On start-up, of the ÄKTA system, a 1 mL Q HP HiTrap anion exchange column (Cytiva Life Sciences, Marlborough, MA) was primed, purged, and equilibrated with 5 column volumes (CV) of wash buffer (25 mM tris, pH 7.6). The protein was manually loaded through the sample injection port to the sample capillary loop (500uL) before injection onto the column. The system was then washed with 5 CV of wash buffer to remove unbound contaminants. A linear gradient of elution buffer (1 M NaCl, 25 mM tris, pH 7.6) up to 70% was used to elute proteins in the sample into 15 mL Falcon tubes, with α-synuclein eluting around 60:40% elution buffer/wash buffer. Proceeding this, a step to 100% elution buffer for 3 CV was used to remove any strongly bound proteins from the column. Column-in-place cleaning was performed with a 20% ethanol buffer and dH_2_O. Positive samples were concentrated using a 3 kDa MWCO filter, further purified with a Superdex 75 size-exclusion column, and equilibrated with buffer (25 mM Tris-HCl, 100 mM NaCl, pH 7.6). The purified sample was concentrated to 5 mg/mL and stored in liquid nitrogen. Protein presence at 14.5 kDa was confirmed by SDS-PAGE and silver staining. The thawed protein was filtered through a 0.22 μm syringe filter with Laemmli buffer, boiled at 95 °C for 5 min, and centrifuged. Gel lanes were loaded with 5 μL PageRuler Plus Ladder and 20 μL of sample. The gel was run at 200 V for 30 min in a Bio-Rad system and stained with a Pierce silver stain kit (Bio-Rad Laboratories, CA). Fibrillation was conducted using a benchtop shaking thermomixer (Thermo Fisher, Waltham, MA) placed in a drying cabinet (LEEC Limited, Nottingham, U.K.), both equilibrated to 37 °C to minimize condensation, which could affect fibril formation.^45^ Three aliquots of protein (250 μL at 5 mg/mL) were thawed at room temperature and placed in a thermomixer, where they were mechanically aggregated at 37 °C and 1000 rpm. Samples were taken on days 1 (D1), 4 (D4), and 7 (D7). A fourth aliquot was designated as Day 0 (D0) and remained untreated. At each time point, the samples were diluted to 0.1 mg/mL in equilibration buffer (25 mM Tris-HCl, pH 7.6), resulting in 12.5 mL of workable PFFs, which were then aliquoted into 1 mL samples and stored in liquid nitrogen.

### Silver Staining

Polyacrylamide gel was washed twice for 5 min in ultrapure water. The gel was then fixed in 30% ethanol:10% acetic acid for 15 min, twice. Next, the gel was washed with 10% ethanol twice for 5 min and ultrapure water twice for 5 min. The gel was sensitized in 50 μL of sensitizer and 25 mL water for 1 min and then washed twice in ultrapure water for 1 min. The gel was incubated with 25 mL of stain and 0.5 mL of enhancer for 30 min, before being washed twice with ultrapure water. The gel was developed with 0.5 mL of enhancer and 25 mL of developer for 2–3 min. When bands became visible, the gel was washed with 5% acetic acid for 10 min and visualized using a ChemiDoc MP imaging system (Bio-Rad Laboratories, CA).

### Negative Staining Electron Microscopy

Purified α-synuclein (10 μL) was adhered to carbon/Formvar-coated copper grids (200 mesh) prepared by glow-discharging for 30 s using a Pelco glow discharge system (Pelco, CA). The sample was then washed with dH_2_O and blotted with Whatman filter paper three times and the sample stained with 2% uranyl acetate for 5 s before blotting. Grids were dried under a lamp before imaging. Negative-stained grids were imaged via an Emsis Xarosa camera in combination with a Hitachi HT7800 TEM and the images were analyzed using ImageJ (National Institute of Health, WA).

### Static and Dynamic Light Scattering

An all-in-one static and dynamic light scattering platform (Uncle, Unchained Laboratories, Pleasanton, CA) was employed to characterize α-synuclein at each time point. Aliquots were removed from liquid nitrogen, gently thawed, and 8.8 μL was injected in triplicate into a Uni platform from each aliquot. The sizing and polydispersity settings were selected, and the system was equilibrated to 37 °C, incubating the samples for 120 s before performing dynamic light scattering measurements. Fresh aliquots were also assessed for sizing using thermal ramp settings with the system equilibrated at 25 °C before ramping to 95 °C at a rate of 1 °C/min. At each degree, 4 acquisitions of 5 s each were collected. Graphs were analyzed using Uncle Data Analysis Client. Data analysis was conducted in RStudio (Posit Team, 2023), using the core “stats” package (R Core Team, 2022) and “ggplot2” (Wickham, 2016).

### Liquid Chromatography–Mass Spectrometry

Samples from each time point were analyzed using both native and denaturing mass spectrometry with an Acquity I-Class UPLC system coupled to a Xevo G2-XS Q-TOF mass spectrometer. (Waters Corporation, U.K.). For intact protein analysis, 50 μg of protein from each time point was desalted using Zeba spin columns (0.5 mL, 7 kDa MWCO) and 7.5 μL injected onto an Acquity UPLC Protein BEH C4 column (300 Å, 1.7 μm, 2.1 mm × 50 mm). The mobile phases were 0.1% formic acid in water (A) and 0.1% formic acid in LC-MS grade acetonitrile (B), with a flow rate of 3 μL/min. The gradient began with 5% B for 1 min, increasing to 60% by 6.5 min, and reaching 95% B briefly before returning to 5% for a total run of 12 min. The analysis, performed in positive electrospray ionization mode (*m*/*z* range 500–4000), used a capillary voltage of 3.00 kV, a cone voltage of 150 V, and a source temperature of 120 °C. Chromatograms were deconvoluted with the MaxEnt1 algorithm in MassLynx to determine protein size in Daltons (Da). For bottom-up MS, 150 μg of total protein was incubated with 0.75 μg of Pierce trypsin protease for 4 h. Samples were processed using HyperSep C18 tips, followed by centrifugation, washing, and elution, then injected onto an Acquity Premier Peptide CSH C18 Column (130 Å, 1.7 μm, 2.1 × 100 mm^2^) at a flow rate of 0.2 mL/min for a 75 min run. Phase B increased from 5% to 100% over the course of the run. Positive electrospray ionization (*m*/*z* range 50–2000) was used with a capillary voltage of 2.80 kV, a cone voltage of 25 V, and a source temperature of 150 °C. Data were analyzed using Progenesis software to align retention times, detect features, and perform statistical analysis of peptide expression changes.

### Raman Spectroscopy Combined with Machine Learning

20 μL of α-synuclein aliquots was dried on a stainless-steel slide at 37 °C to create a “coffee-ring” effect.^46^ The samples were analyzed using an inVia confocal Raman microscope (Renishaw, Gloucestershire, U.K.). Calibration was performed by using an internal silicon reference before each set of measurements. The edge of each drop was examined with a 785 nm laser, capturing 10 spectra per time point, with each spectrum representing an average of 5, 1 min accumulations at 100% laser power. Cosmic ray removal and spline baseline subtraction were applied.

Spectral data were processed in Microsoft Excel (Microsoft, Redmond, WA) and analyzed in RStudio (2024 Posit Software, PBC, formerly RStudio, PBC). Raw data was plotted to identify large-scale variations within the data set. Standard normal variate (SNV) normalization was performed using the RamanMP RStudio package.^47,48^ This package adjusts each intensity value by subtracting the mean intensity of the spectrum and dividing by the standard deviation of the spectrum. This transforms the data into a standardized scale, removing multiplicative and additive scatter effects. Once normalized, principal component analysis (PCA) was conducted to reduce data dimensionality data. A threshold of 80% was established to identify the most significant principal components (PCs). The first 3 PCs, explaining 89% of the variation, were retained for further analysis. Loading plots identified nine key wavenumbers, with additional wavenumbers found through interrogation of overlays and heatmaps. Statistical analyses were performed to evaluate group differences. ANOVA was applied, followed by Tukey’s HSD post hoc test for normally distributed data. For non-normally distributed data, the Kruskal–Wallis test was used, followed by Dunn’s test with Bonferroni correction. Significance thresholds were set at *p* < 0.05, resulting in the exclusion of four nonsignificant wavenumbers from these analyses. Unsupervised learning using Uniform Manifold Approximation and Projection (UMAP) was also performed to identify nonlinear relationships and visualize group separations.^49^ UMAP analysis was repeated across 10 random seeds to ensure reproducibility of results, with averaged outputs used for visualization.

## Results

### Structural and Size Changes of α-Synuclein during Fibrillation

**Figure 2:**
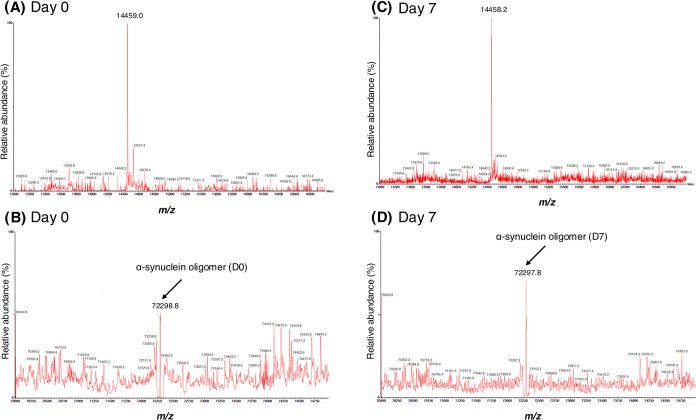
(A) Intact LC-MS/MS analysis confirmed the presence of α-synuclein, with a detected protein mass of 14 459 Da on D0 of the assay. (B) Mass spectra from D0 show a small peak corresponding to the pentameric weight of α-synuclein. (C) On D7, a similar mass was detected, indicating the continued presence of monomeric α-synuclein throughout the assay. (D) Spectra from D7 display a larger peak at the pentameric weight of α-synuclein, suggesting the formation of larger protein aggregates, consistent with fibrillation progression over time.

Silver staining confirmed the successful purification of α-synuclein, revealing a single distinct band at ∼15 kDa (Figure 2A). TEM was then employed to visualize the fibrillation process. In the early stages of the fibrillation assay, microscopy revealed small, unstructured aggregates of approximately 30–50 nm in size. As fibrillation progressed, larger aggregates were observed, reaching sizes of up to 100 nm, suggesting initial fibril formation. In the later stages of the assay, complex structures with prominent fibril-like features emerged, measuring up to 350 nm (Figure 2B). Quantitative analysis of fibril sizes revealed a significant increase in fibril size from D0 to D1 (*p* < 0.0001), indicating a rapid aggregation phase. This was followed by a plateau in fibril size from D1 to D4 (p = 0.98), suggesting a lag phase in fibrillation. A significant increase in size was again observed between D4 and D7 (*p* < 0.01). By D7, the width distribution of fibrils displayed considerable variability, suggesting that not all proteins had aggregated into uniform structures (Figure 2C). Static and dynamic light scattering analyses demonstrated a significant increase in the size of α-synuclein aggregates over the 14-day assay. The polydispersity of the samples rose dramatically, from 29.28 nm on D0 to 100.52 nm by D7 (*p* < 0.01). Additionally, the Z-average size showed a marked increase, rising from 54.77 nm on D0 to 176.86 nm on D7 (*p* < 0.001). These results indicate a transition from predominantly small aggregates to larger, more heterogeneous structures throughout the fibrillation assay (Figure 2D).

**Figure 3:**
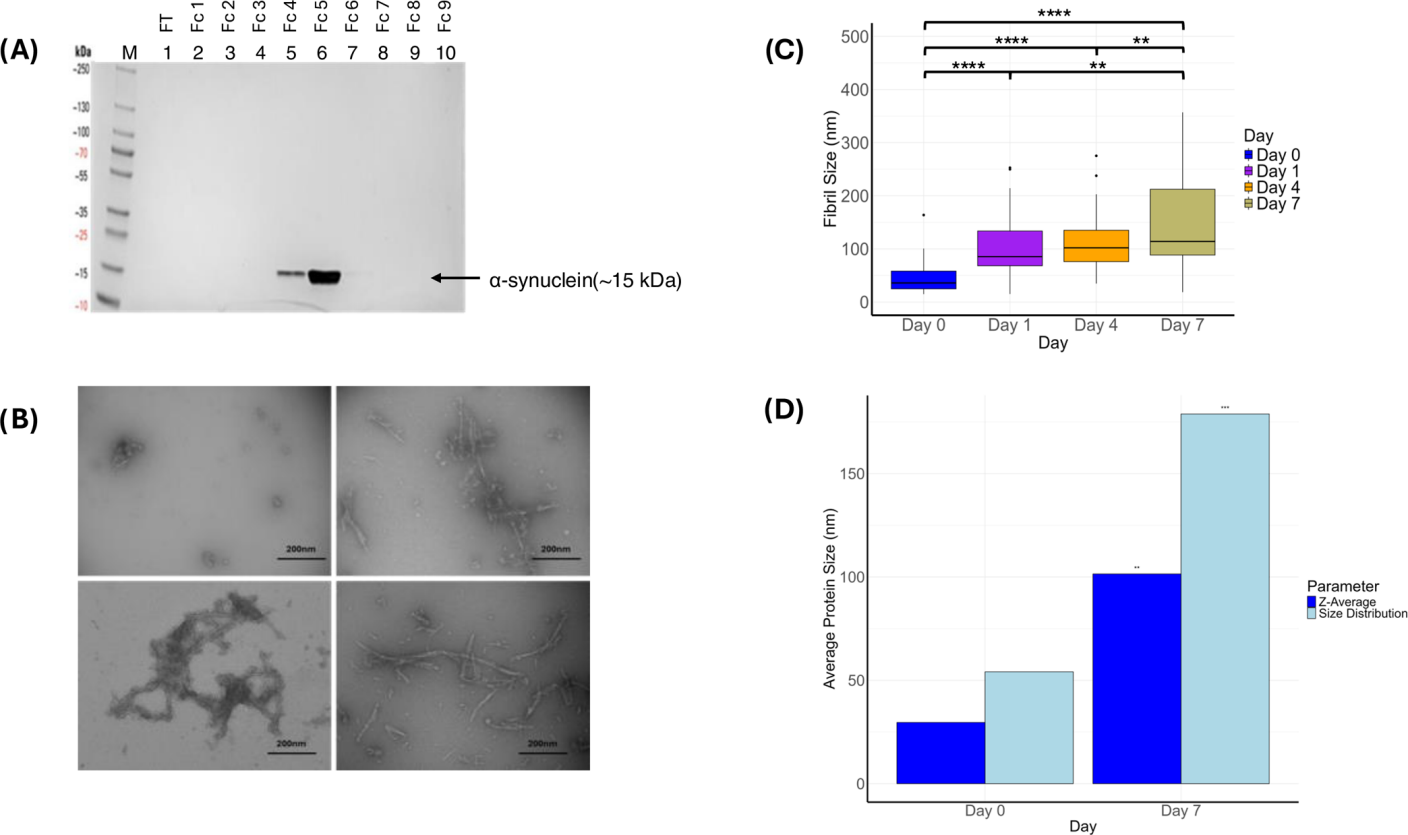
(A) SDS-PAGE analysis of purified α-synuclein fractions followed by silver staining, revealing a pure ∼15 kDa protein. (M) Markers, (FT) Flowthrough. (B) Transmission electron microscopy (TEM) images from negative staining, showing the progression of α-synuclein aggregation: small monomers on D0 (top left), oligomers on D1 (top right), and larger fibrillar structures on D4 (bottom left) and D7 (bottom right). (C) Box and whisker plot depicting the median fibril size distribution over the 7-day α-synuclein fibrillation assay. Fibril size increases over time, with a small lag phase between D1 and D4 (*p < 0.05, **p < 0.01, ***p < 0.001, ****p < 0.0001). (D) Static and dynamic light scattering data showing the average protein size increase from D0 to D7. Both size distribution and Z-average measurements are significantly higher on D7 compared to D0, indicating protein fibrillation (*p < 0.05, **p < 0.01, ***p < 0.001, ****p < 0.0001).

Intact LC-MS/MS analysis confirmed that 39 out of 45 identified peptides aligned specifically with α-synuclein, demonstrating high specificity in its detection. Additionally, 10 peptides were associated with β-synuclein (4 unique), suggesting close homology within the synuclein family. The remaining six peptides were linked to unrelated proteins, including guanylate cyclase (2 unique), GPALPP motif-containing protein 1 (2 unique), transmembrane protein 50B (1 unique), and serine/threonine-protein kinase SMG1 (1 unique). These findings indicate that while most peptides are specific to synucleins, the presence of unrelated proteins likely represents minor contaminants or methodological artifacts. A protein mass of 14 459 Da on D1 of the assay (Figure 3A) was identified, which was also detected on D7, indicating the consistent presence of α-synuclein throughout the assay (Figure 3B). During the analysis, a small peak corresponding to pentamers was observed on D1 (Figure 3C), whereas the spectra on D7 showed a larger peak, suggesting the formation of larger protein aggregates (Figure 3D). Given that the protein was expressed in *E. coli*, which lacks the cellular machinery for post-translational modifications (PTMs), the analysis confirmed the absence of any PTMs on the synuclein peptides, as expected.^50^ This is important, as it suggests that the observed aggregation pattern can be attributed to intrinsic properties of α-synuclein rather than modifications.

### Spectral Analysis of α-Synuclein Fibrillation

**Figure 4:**
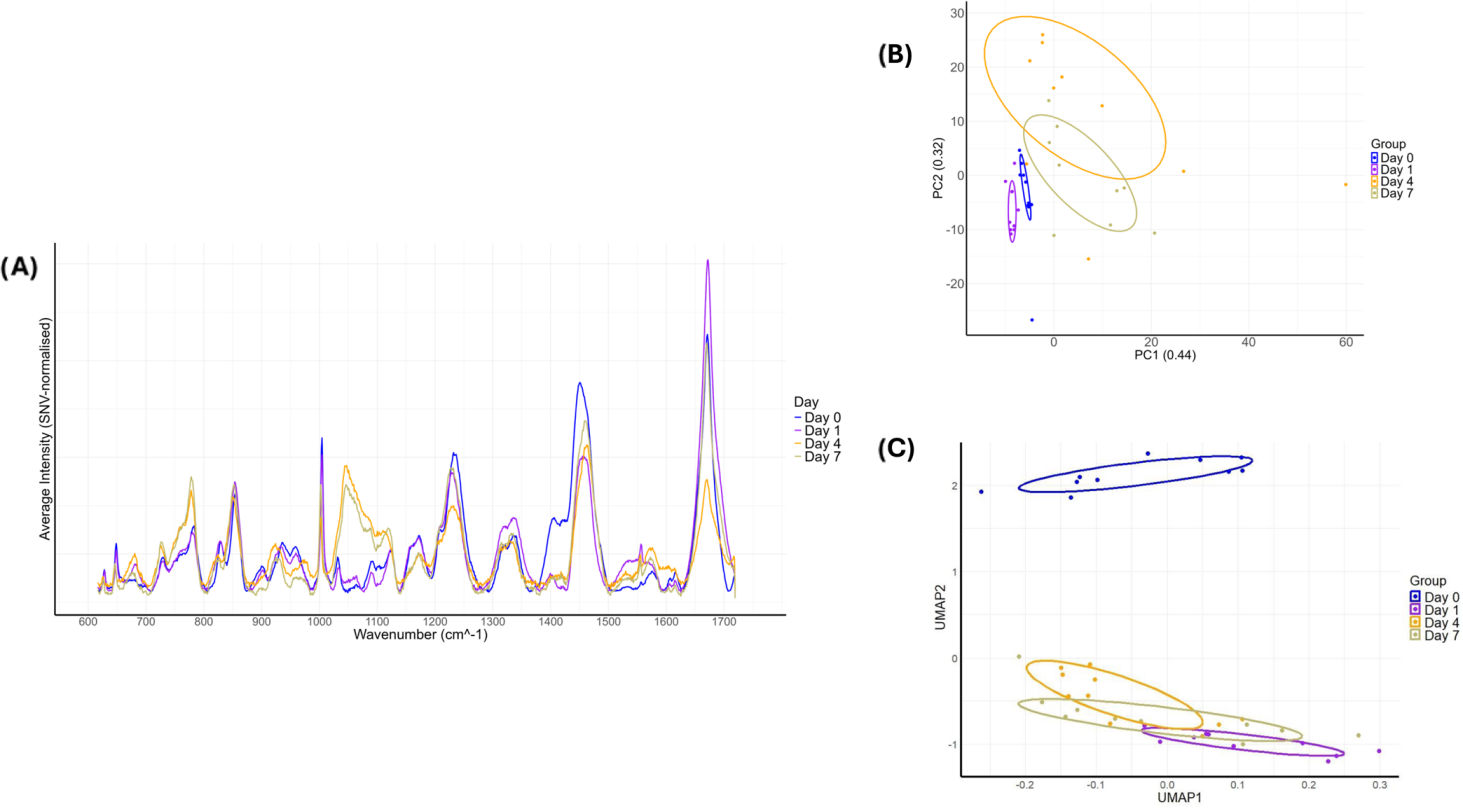
(A) Plot showing the Raman spectral intensities throughout the fibrillation assay. The intensities drop after D0, with a prominent peak observed between 1045 and 1100 cm^–1^ from D4 to D7. (B) Principal component analysis (PCA) plot using PC1 and PC2 to illustrate the largest variations between samples. D4 and D7 cluster separately from D0 and D1, showing overlap between D4 and D7. (C) Ten Uniform Manifold Approximation and Projection (UMAP) analyses were averaged to generate a plot that preserves global and local structures. The UMAP accurately clusters D0 away from D1, D4, and D7 but fails to distinguish between D4 and D7.

Raman spectral analysis of α-synuclein during the fibrillation process revealed distinct changes observed at various time points (D0, 1, 4, and 7) correlated with the dynamic transitions of α-synuclein from monomeric forms to oligomeric aggregates and ultimately to fibrillar structures (Figure 4A). Specifically, the progressive decrease in intensity of peaks associated with α-helical structures on D1–D7 indicated a structural transition toward β-sheet-rich conformations which were increased, characteristic of late-stage fibrillation. Notably, the D0 samples exhibited significantly higher intensities compared to the samples from D1, D4, and D7. This trend suggests a decrease in Raman activity as the protein aggregates into a more disordered structure during the fibrillation process. Throughout the 7-day fibrillation assay, a total of 16 wavenumbers, each corresponding to molecular Raman vibrational modes, were identified as significantly influential (*p* < 0.05) (Table 1). On D1, there was a notable increase in peak intensities at 1303 and 1330 cm^–1^, which are indicative of α-helical structures.^51^ Spectral shifts in the range of 1572–1576 cm^–1^, shifting to 1556 cm^–1^ on D1, reflect N–H bending and C–H stretching in protein backbones, likely indicating dynamic secondary structure changes within the sample.^52^ This suggests that the protein adopts helical conformations early in the fibrillation process. However, by D4 and D7, the intensities of these peaks decreased, coinciding with a significant increase in the intensity of the peaks in the 1045–1105 cm^–1^ range, which are associated with β-sheet formation.^42^ The presence of an increased peak at 1675 cm^–1^ on D1 further suggested a mix of secondary structures,^53^ which diminished by D4. These observations imply a dynamic structural transition, where α-helical structures formed initially transition to β-sheet structures as fibrillation progresses. The emergence of a prominent peak in the 1045–1100 cm^–1^ region on D4 and D7 is particularly significant as it may indicate a marker of late-stage fibrillation, correlating with the transformation into a more structured aggregate. Notably, a decrease in intensity at 1625 cm^–1^, associated with β-sheet hydrogen bonding,^42,54^ was observed, suggesting that these hydrogen bonds may either be transient or become undetectable as the protein adopts a more stable fibrillar form. This decrease in intensity could reflect a reorganization or shielding of hydrogen bonds as the protein transitions into its stable fibrillar state, making these bonding patterns less accessible to spectroscopic detection^55^ (Figure 4A).

**Table 1:** Summary of Changes in Influential Wavenumbers Identified through Analysis of Spectral Overlays and Machine Learning Analysis of Loading Plots^a^

wavenumber (cm^–1^)	allocation	D1 changes	D4 changes	D7 changes
650	tyrosine C–C twisting of aromatic ring		↓	↓
725	proline			↑
780	alanine			↑
830	tyrosine-ring-breathing mode and out-of-plane vibration of the benzene ring			↓
900	vCC		↓	↓
950	vCC		↓	↓
1045	skeletal C–C/C–N stretching in β-sheets		↑	↑
1175	C–C stretching in tyrosine’s aromatic ring		↓	↓
1303	α-helices	↑	↑	
1330	α-helices	↑	↑	
1406	COO^–^ symmetric stretch	↓	↓	↓
1420	C–H bond scissoring	↓	↓	↓
1445	CH_2_ protein bonds	↓	↓	↓
1493	C–H and N–H bending	↓		↓
1625	H-bonding in β-sheet structures			↓
1675	combination of α-helices and β-sheets	↑	↓	

a Each wavenumber showed a statistically significant increase (↑) or decrease (↓) in spectral intensity at D1, D4, or D7 of the fibrillation assay, when compared to D0 protein samples.

Accompanying the dynamic transitions of α-synuclein from monomers to oligomers and fibrils, prominent changes in amino acid residues were observed throughout the fibrillation process. Decreases in intensity were found at 1406 cm^–1^, related to COO^–^ symmetric stretching,^43^ across D1, D4, and D7. This finding suggests that glutamic acid^56^ is involved in the early stages of fibrillation, potentially playing a role in initiating α-synuclein aggregation or being sequestered during the initial aggregation process. In contrast, peaks at 725 and 780 cm^–1^, indicative of proline and alanine, respectively,^57,58^ increased in intensity during the later stages, suggesting that these residues become more accessible as the protein transitions to a fibrillar state, possibly because of the release of the hydrophobic core domains of the protein. Interestingly, Raman peaks associated with tyrosine exhibited significant decreases throughout the assay. Specifically, intensities at 650 and 1175 cm^–1^^52,59,60^ diminished by D4 and D7, while intensities at 830 cm^–1^^42,52,60^ decreased on D7. This multistage decrease suggests that tyrosine residues may have complex roles in α-synuclein fibrillation, influencing both structural integrity and intermolecular interactions as fibrillation progresses. Significant reductions were also observed at D4 and/or D7 for peaks relating to C–C stretching (900, 950 cm^–1^),^60^ C–H rocking (1420 cm^–1^),^52^ CH_2_ protein bonds (1445 cm^–1^),^61^ and C–H and N–H bending (1493 cm^–1^).^62^ A spectral shift was identified on D0 at 1448 cm^–1^, which shifted to the 1455–1465 cm^–1^ range in later days. This peak is associated with C–H deformations, typically from proteinaceous amino acid side chains.^52^ The shift on D0 likely reflects overall structural changes within the protein, indicating the transition from an unstructured monomer to a more structured oligomer. These changes in generic protein vibrations indicate that the protein has transitioned to a less Raman-active structure, reflecting the formation of stable aggregates (Figure 4A).

### Dimensionality Reduction and Clustering Analysis of α-Synuclein Fibrillation Stages

To investigate the structural changes in α-synuclein during fibrillation, we performed PCA and UMAP analyses on the data. The PCA plot (Figure 4B) showed that the first two principal components (PC1:44%, PC2:32%) captured a substantial portion of the variance within the data. Clear clustering of D0 and D1 samples indicated distinct changes as the protein transitioned from monomeric to oligomeric forms in the early stages. In contrast, D4 and D7 samples clustered closely together, suggesting that similar structural changes occur during these time points, likely corresponding to the transition toward a β-sheet-rich fibrillar structure. In addition to PCA, a UMAP analysis was performed to explore nonlinear patterns. The UMAP plot corroborated the distinct separation between D0 and the subsequent time points, emphasizing the profound structural changes occurring in the early stages of fibrillation. However, UMAP analysis did not differentiate between D1, D4, and D7, suggesting that similar and uniform changes are occurring during these later stages. While PCA effectively captured the linear relationships and distinct stages of α-synuclein fibrillation, UMAP did not differentiate between the later time points, reinforcing the idea that the data exhibits linear patterns (Figure 4C).

## Discussion

In this study, we employed a combination of techniques, including Raman spectroscopy with machine learning, to investigate the biomolecular changes during α-synuclein fibrillation. Raman spectroscopy revealed key structural alterations, particularly a shift from an α-helical to a β-sheet conformation, along with changes in specific amino acid residues, specifically those of a more hydrophilic origin such as tyrosine, glutamic acid, or the more hydrophobic alanine and proline. These findings were corroborated by complementary analyses using LC-MS/MS, light scattering, and negative staining TEM, which aligned with the observed biochemical transformations. Negative staining TEM revealed clear fibril growth, showing a rapid increase in size from D0 to D1, followed by a lag phase and subsequent significant growth from D4 to D7. This pattern corresponded to the formation of stable β-sheet structures seen in the Raman spectra. Similarly, static and dynamic light scattering demonstrated an increase in particle size from D0 to D7, reflecting the transition to larger aggregates, consistent with the β-sheet shift detected by Raman spectroscopy. PCA and UMAP analyses of the Raman data showed distinct clustering of early-stage (D0 and D1) and later-stage (D4 and D7) samples, indicating early structural transition from monomers to oligomers, followed by stabilization of β-sheet-rich aggregates.

This study presents a novel approach to assessing α-synuclein aggregation by integrating Raman spectroscopy with an in-house machine learning pipeline, enabling the identification of unique spectral features and amino acid changes throughout the aggregation process. Previous studies employing Raman spectroscopy to investigate α-synuclein have predominantly focused on the 1670 cm^–1^ peak as a marker for β-sheet presence.^43,63,64^ While valuable, this offered a limited perspective by excluding much of the biological spectrum. In contrast, this study used machine learning to analyze the entire Raman spectrum, identifying 16 significant peak changes, including a novel 1045–1100 cm^–1^ peak, which has not previously been associated with α-synuclein fibrillation. These findings provide enhanced structural insights and reveal biochemical changes, including several novel amino-acid-specific shifts linked to Raman intensity fluctuations, throughout the aggregation process.^64^ While bioinformatics pipelines, such as dimensionality reduction techniques have been applied to analyze complex multiomic data sets, in α-synuclein in murine models,^65^ their application to the study of α-synuclein aggregation remains limited.

The misfolding and aggregation of α-synuclein monomers during fibrillation followed a structural transition from α-helical conformations to β-sheet-enriched insoluble forms. In the early stages of fibrillation, particularly on D1, Raman peaks at 1303 and 1330 cm^–1^, associated with α-helical structures, confirm that α-synuclein initially adopts an α-helical conformation.^51^ α-helical tetramers of α-synuclein, thought to represent some physiological forms of the protein, have been proposed to delay the onset of fibrillation, aligning with the observed increase in α-helical peaks during the early lag phase of fibrillation.^66^ However, as the fibrillation process progressed, the α-helical structure peaks diminished. By D4 and 7, the intensity of the α-helical peaks at 1303 and 1330 cm^–1^ significantly decreased, coinciding with a marked increase in the 1045–1100 cm^–1^ range, indicative of β-sheet formation.^43^ The transformation from α-helical to β-sheet structures is also reflected in changes in other Raman peaks. On D1, a peak at 1675 cm^–1^ indicated the presence of the “misfolded” amide I mode,^67^ suggesting a mixture of secondary structures and dynamic transitions between conformations. By D4, this peak diminished, signaling the stabilization of β-sheet structures as aggregation progressed.^53^ Similarly, a significant decline in the intensity of the 1625 cm^–1^ peak by D7 indicated a reduction in hydrogen bonding within β-sheet structures, suggesting these bonds become transient or less accessible as the protein adopts a more stable, aggregated form.^54,68^ Additionally, a reduction in C–C stretching and CH_2_ bonds present in amino acid residues suggested a more ordered protein structure as the fibrillation progresses. This observation aligns with the overall decline in spectral intensity from D0 to D7 further supporting the notion of increased protein ordering during fibrillation. Interestingly, the prominence of the β-sheet peak in the 1045–1100 cm^–1^ region, a significant marker of late-stage fibrillation, has not been extensively reported in pathological studies of α-synuclein. However, previously, amide-III shifts around 1230 cm^–1^ and β-sheet peaks at 1667 cm^–1^ have been reported.^43^

Our study also uncovered the involvement of key amino acids in the conformational changes of α-synuclein during fibrillation. Peaks related to tyrosine residues decreased as fibrillation progressed, highlighting a prominent role in intramolecular interactions or incorporation into fibrils. Raman intensities at 650 and 1175 cm^–1^ decreased on D4 and D7, and the intensity at 830 cm^–1^ decreased on D7. Dual peaks at 830 and 850 cm^–1^ have been associated with Fermi resonance doublet of tyrosine; highlighting the ring-breathing mode and out-of-plane vibration of the tyrosine benzene ring.^43^ A peak at 650 cm^–1^ has been identified as C–C twisting in the tyrosine aromatic ring,^52,59^ while a peak at 1175 cm^–1^ has been associated with the C–C stretching in the tyrosine aromatic ring.^60^ The nature and location of tyrosine residues influence aggregation, with distinct regions playing specific roles depending on the protein’s conformation.^28^ On interaction between two α-synuclein monomers, C-terminal tyrosines react first, while the compact structure hinders further reactions.^28^ Without an N-terminal tyrosine (e.g., Tyr39), dimers form initially, followed by larger aggregates like tetramers and hexamers.^69,70^ Tyrosines 39 and 125 are less reactive, resulting in fewer higher-order aggregates in variants with only these residues.^28^ Unfolding α-synuclein with guanidine hydrochloride exposes C-terminal tyrosines, eliminating the need for an N-terminal tyrosine for aggregation.^28^ Notable decreases in tyrosine-related Raman peaks at 650 and 1175 cm^–1^ by D4 and D7, as well as a decrease at 830 cm^–1^ by D7, suggest that such residues may undergo modifications, such as oxidation,^71^ oxidative cross-linking^72^ or become incorporated into the fibrillar structure, limiting their detection. Cross-linking initiates primary nucleation, triggering conformational shifts that stabilize α-synuclein dimers, forming seeds for toxic oligomers.^73^ The significance of tyrosine in α-synuclein aggregation is illustrated by the effects of various mutations in the context of disease pathophysiology. The Tyr136Ala mutation, for example, is known to delay fibril formation compared to the wild type.^74^

A peak at 780 cm^–1^, indicating alanine presence, increased on D4 and D7.^75^ α-synuclein contains 15 alanine residues, each contributing to the fibrillation process in an under-characterized manner. This complexity makes it challenging to attribute intensity changes to a single residue. However, collectively, these residues participate in forming hydrophobic cores that may play a role in protein aggregation.^76^ Mutations in specific alanine residues have been linked to familial PD. For example, the alanine-to-threonine missense mutation at position 53 (A53T) has long been associated with early onset PD,^77^ while the alanine-to-proline mutation at residue 30 is thought to disrupt interactions between the N- and C-terminal domains, promoting fibrillation and contributing to familial PD.^78^ This suggests an important role of alanine in early monomer stabilization. However, as monomers interact during aggregation, the vibrational potential of alanine residues may decrease, leading to reduced peak intensities. Once aggregation occurs and these interactions are disrupted, certain alanine residues could become more vibrationally active, causing the observed increase in alanine peaks in the Raman spectrum in the latter stages of the assay. Alanine-76, for instance, resides in an 11-amino-acid region that forms the hydrophobic core of α-synuclein aggregates, emphasizing the role of alanine in fibrillation.^79^

A decrease in intensity at 1406 cm^–1^, a peak related to COO^–^ stretching, likely from glutamic acid residues, was observed on D1, D4, and D7.^80^ Glutamic acid is known to inhibit β-sheet formation, with Glu-46 involved in C–N-terminal interactions that stabilize the monomeric form of α-synuclein.^13^ This stabilization is likely evident in Raman spectra as a stronger intensity peak on D0 of the fibrillation assay. However, as aggregation begins, the intensity at 1406 cm^–1^ decreases at D1. Cryo-electron microscopy studies have suggested that during nucleation, Glu-46 forms a salt bridge with Lys-80 at the core of growing α-synuclein fibrils.^81^ This structural change likely prevents Glu-46 from Raman detection, which may also explain the reduced peak intensities observed. The E46K mutation, where glutamic acid is substituted with lysine in a conserved region of the protein, has been linked to familial PD and is likely to significantly disrupt protein function.^82,83^ Additionally, the mutation of Glu-83 to alanine has been shown to have an inhibitory role in amyloid formation.^84^ Recently, the E83Q mutation has been associated with DLB, the first identified in the NAC domain of α-synuclein.^85^

On D7 an increase is seen at 725 cm^–1^, commonly attributed to proline residues.^57^ The C-terminal domain contains all proline residues in α-synuclein, which act as helical breakers by facilitating interactions between the C-terminal domain and the NAC, inhibiting the formation of α-helices.^76^ These interactions could impact intramolecular vibrations of proline residues, producing minimal intensities at the relevant Raman peaks. The subsequent increase in proline peak intensities on D7 coincides with β-sheet formation. This suggests that as β-sheets form, proline interactions are disrupted, making the residues accessible for Raman detection and increasing peak intensities, and not incorporated into β-sheets, rendering them Raman inactive. Proline residues do not participate in β-sheet formation because their pyrrolidine ring hinders the ability to form hydrogen bonds.^86^ This means proline residues are more likely to be found exposed on the edges of the β-sheet or in linker regions,^87^ which are still readily available to Raman scrutiny, subsequently leading to intensity increases at relevant peaks.

Unsupervised PCA (Figure 4B), UMAP (Figure 4C), and supervised learning were employed to assess the potential between time points in the fibrillation assay, providing insight into distinct stages of α-synuclein aggregation. The distinct patterns observed in each analysis highlight how these methods process and retain information differently. UMAP, which is effective in identifying complex patterns and localized clusters, captured more nuanced groupings, while PCA, suited to linear relationships, provided a clearer representation of broader structural transitions. All methods consistently identified distinct clustering at D0. PCA characterized D0 as a small ellipsis, indicating a homogeneous population of monomeric α-synuclein, supported by static and dynamic light scattering analyses (Figure 3D), which confirmed a monodisperse population at this time point. UMAP also revealed a significant separation between D0 and D1, with a large distance between these time points, corroborated by the high variance explained by the first two components, indicating dissimilarity. This separation, coupled with an increase in α-helical peak intensities, suggesting the formation of tetrameric α-synuclein.^88^ The transition from a monomeric to tetrameric form notably affected the Raman spectra, as seen in the rapid decrease in Raman intensities from D0 to D1, which likely contributed to the large separation on the UMAP plots. These findings hold significant promise in Lewy body disease diagnostics as a template for protein aggregation assays, such as combining α-synuclein seed amplification assays with Raman spectroscopy and machine learning techniques. By improving sensitivity and accuracy in detecting unique aggregation signatures, this method could aid the development of noninvasive, rapid diagnostic frameworks for diseases driven by protein misfolding, where early and precise biomarker detection is critical. Through the capture of distinct structural signatures, such as changes in amino acid residues and their correlation with pathological states, integrating Raman spectroscopy with machine learning provides a robust and powerful tool for characterizing the dynamic processes of protein aggregation.

We acknowledge the limitations of this study, including the absence of circular dichroism (CD) spectroscopy to assess the secondary structure changes in α-synuclein. While complementary techniques such as static and light scattering, LC-MS, and negative staining TEM were employed, collectively providing a comprehensive understanding of the aggregation process, future studies will incorporate CD spectroscopy to validate the trends observed in Raman spectral intensity peaks by comparing them with the quantitative secondary structure content.

## Conclusions

In conclusion, we used Raman spectroscopy combined with machine learning to identify distinct stages in α-synuclein fibrillation, providing valuable insights into key conformational changes and molecular interactions. Our findings demonstrated an initial increase in α-helical peaks, indicative of tetramer formation, followed by a marked shift toward β-sheet-rich structures as fibrillation progressed. This structural transition was accompanied by changes in specific amino-acid-associated peaks, particularly those related to tyrosine, alanine, glutamic acid, and proline residues. Complementary LC-MS/MS, TEM, and light scattering analyses confirmed the rapid initial growth of fibrils, a subsequent lag phase, and a significant increase in particle size and stability associated with β-sheet formation at later stages. Additionally, PCA and UMAP analyses further highlighted distinct aggregation stages, with clustering patterns indicating a clear transition from monomeric to oligomeric forms and subsequent β-sheet stabilization. By tracking molecular-level changes during fibrillation, our approach highlights the specific contributions of amino acid residues and secondary structure transitions in α-synuclein misfolding, providing a useful framework for further exploration of protein aggregation in Lewy body disease diagnostics.
